# Gender identity and sexual orientation in Austrian adolescents

**DOI:** 10.1371/journal.pgph.0004279

**Published:** 2025-02-21

**Authors:** Lukas Teufl, Friedrich Teutsch, Roman Winkler

**Affiliations:** Austrian National Public Health Institute (Gesundheit Österreich GmbH), Vienna, Austria; UNAM: Universidad Nacional Autonoma de Mexico, MEXICO

## Abstract

In many European countries, representative data on the proportion of lesbian, gay, bisexual, transgender, and queer (LGBTQ) adolescents are still lacking. Therefore, this study used data of 9,295 adolescents (11 to 17 years) who participated in two nationwide surveys (Health Behaviour in School-aged Children Study and Austrian Health Study on Apprentices) and investigated answers regarding gender identity and sexual orientation. Additionally, age- and gender-specific patterns and mocking answers given were quantitatively and qualitatively analyzed. Results show that 0.8% of Austrian adolescents identified with a gender other than female or male and 10.5% with a sexual orientation other than heterosexual, with other sexual orientations being more common in girls (14%) than boys (4%). Only five adolescents were unsure about their gender identity and every twentieth adolescent did not identify with a sexual orientation yet. Most adolescents took the questionnaire seriously, with only 82 (0.8%) mocking answers given. These mocking answers were classified in seven groups, with Popular Culture and Memes being the largest group. This study makes a significant contribution to the collection of data on gender identity and sexual orientation of (Austrian) adolescents.

## Introduction

In the last decades, many European countries emphasized more awareness of specific problems and needs of LGBTQ people which resulted in political action plans. These action plans typically facilitate projects and initiatives in health care, education, the workplace, laws and legal protection, public service, sports, tourism, media, and science [1]. Many actions also focus on LGBTQ youth aiming for a healthier and less threatening and discriminating context in their everyday lives. In the best case, these actions are based on monitoring data. North America has a longer history than Europe of studies on prevalences of LGBTQ people in the total population. Also, from 2013 onwards, more studies also focused on adolescents. Due to sampling techniques, different age groups and operationalization of gender identity, the prevalences found vary. For example, for transgender adolescents the estimated prevalences ranged from 1.3% for 11- to 14-year-olds [2] and 1.7% to 3.8% for 14- to 17-year-olds [3,4]. Further, some studies also concentrated on nonbinary identities resulting in comparable prevalence estimates between 1.0% and 3.8% of 11- to 19-year-olds [4,5,6]. Importantly, some adolescents were unsure about their gender identity, ranging from 1.0% up to 2.3% [5,7]. North American studies of sexual orientations other than heterosexual also found variations in the estimated prevalences ranging from 1.3% to 6.0% for homosexual and 2.1% to 8.4% for bisexual 11- to 18-year olds with higher prevalences for girls compared to boys [2,5,7].

In Europe, demographic studies on prevalences of gender identities and sexual orientations are still scarce and the estimates seem to differ from North America. Among these studies, prevalences for transgender adolescents varied between 0.6% for under 21-year-olds in Finland [8] to 1.6% for non-cisgender 11- to 17-year-olds in Germany and Netherlands [9,10]. These studies also analyzed prevalences of nonbinary adolescents, ranging from 1.1% to 4.6% in these countries [8,10,11]. Estimates for sexual orientations other than heterosexual were studied in Germany, yielding estimates from 2% to 3% for homosexual and 3% to 8% for bisexual adolescents [12]. There is also a large-scale survey [13] conducted in 27 countries worldwide that also included 10 European countries, but not Austria. This study focused on prevalences of gender identities and sexual orientations for Gen Z (14- to 20-year olds) and Millenials. They found that overall, 4% of Gen Z identified themselves as transgender, 4% as nonbinary, 4% as homosexual, 9% as bisexual and 2% as asexual.

The lack of European studies may stem from the relatively new focus on topics concerning LGBTQ youth. However, a second reason might be found in uncertainties of scientists concerning the appropriate age to ask adolescents about their gender identity and sexual orientation. Findings from research using perspectives in developmental psychology provide important insights on this matter.

Regarding gender identity an exploratory interview study [14] on transgender children and adolescents in Switzerland found three different developmental pathways for becoming aware of one’s own non-cis-gender identity. These include, (1) Affirmed: Experiences and perceptions that the assigned gender at birth does not feel right occur from a very early age, while exploration and affirmation start in early to middle childhood; (2) Silent: Feelings of gender dissonance start very early again, but these feelings are hidden from family and/or caretakers, therefore exploration and affirmation start around the onset of puberty. (3) Neutral: Feelings of dissonance and exploration start with the onset of puberty, while beforehand one’s gender was hardly questioned. An Italian study [15] on 197 transgender and nonbinary youth analysed when they first had the feeling, that their gender might be different from the sex they were assigned at birth and when they identified as trans or nonbinary. Transgender youth reached both milestones earlier than nonbinary youth. On average, they reached the first milestone with 10 years vs. 12 years and the second one with 20 years versus 23 years. However, standard deviations were huge with 6 to 8 years, which might stem from the sample size and/or the actual high variations in reaching these milestones. Overall, the onset of puberty, typically starting at 10 to 12 years of age [16], seems to be a common point for transgender adolescents to start exploring or intensify the exploring of their gender dissonance.

A meta-analysis on the developmental milestones of sexual orientations included 30 studies conducted in the United States [17]. Among the identified milestones (e.g., Attraction, Questioning One’s Orientation, Self-Identifying, Sexual Activity, Coming Out, Romantic Relationship), *Self-Identifying* may be a key milestone for measuring sexual orientation, because as soon as a person identifies with a certain sexual orientation, reporting about it on a more general level is also possible. On average, participants achieved this milestone when they were 17 years old; the variance was large, however, with the 95% confidence interval reaching from 11 to 24 years. The included studies also covered participants born as early as 1965, when life circumstances for LGBTQ people in the United States were more threatening and oppressive, and their sexual orientations were oftentimes kept a secret. Therefore, the results cannot be transferred to the current generation of LGBTQ youth.

On closer inspection, one included study [18] focussing on participants born in 1988 or later found that, on average, cisgender boys and cisgender girls achieved the milestone of Self-Identifying at the age of 13 and 15, respectively. This underlines two important issues: (1) adolescents nowadays develop their sexual orientation faster than preceding generations and (2) Self-Identifying is a gender-specific process.

The identification process of asexuality, which may be defined as the lack of sexual attraction to others or a low or absent interest for **sexual** activity, is following a similar pattern [19,20]. According to McInroy et al. (2021) asexual 14- to 29-year-olds first thought about being asexual on average when they were 13 years and knew for sure when they were 15 years. However, standard deviations were large with 3 years.

In summary, we hypothesize that most adolescents should be able to report on their diverse gender identity starting at the age of 10 to 12 years and on their sexual orientation starting at the age of 13 to 15 years.

Discrimination and hostile attitudes towards LGBTQ people are still found, even in younger generations, [e.g. 21]. In contemporary popular media, like video games, social media, music videos as well as TV shows and movies, LGBTQ people are presented ambiguously. For example, certain TV shows actively decide to include non-stereotypical LGBTQ characters to reduce stigmatization whereas in social media hostile memes (= an image or piece of text that is typically humorous in nature, that is copied and shared rapidly by internet users) mocking LGBTQ-related topics are shared.

This ambiguity may also lead to different perceptions of scientific questionnaires asking about gender identity and sexual orientation, especially among adolescents. They may be received well by some adolescents who may appreciate the openness towards and importance of including various gender identities and sexual orientations in studies. On the other hand, surveys with questions concerning sexual orientation and gender identity may also provoke reactance and aggression in other young people who have hostile attitudes towards LGBTQ people. In scientific investigations, this may lead to mocking, hostile or missing answers. Even though these answers may reflect attitudes towards LGBTQ people, the data will not be usable for its intended purpose. This may be especially risky in surveys and panel studies when gender (identity) is an important group variable for many other research areas in the study. We do not know how Austrian adolescents react to these questions or how many of them do not take them seriously.

The present study relied on two Austrian survey studies on adolescent pupils and apprentices aged 11 to 17 years. For this study, two items on gender identity and sexual orientation were investigated, which allowed for further specifications. The following research questions guided this study:

(1) Which gender identities do adolescents identify with and how are they distributed in Austria? Are there age- or group-specific (pupils vs. apprentices) variations?
(2) Which sexual orientations do adolescents identify with and how are they distributed in Austria? Are there age-, group- (pupils vs. apprentices) or gender-specific variations?
(3) How many and which mocking answers were given? What are the common topics of these answers?

## Methods

### Ethics statement

An ethics and appraisal committee, with representatives of the Ministry of Education, the Ministry of Health, the teacher associations, the parent associations, the Austrian Economic Chamber and the Chamber of Labour had scrutinized the study design as well as the survey questionnaire before data gathering started. Following the positive evaluation, the Ministry of Education published an enactment [ID: 2021-0.669.055] to inform all corresponding schools about the possibility to participate in these studies. Participation of schools, students and apprentices was also voluntary. All parents of adolescents younger than 18 years received a letter with information about the study with the right to refuse the participation of their children.

### Sample

This study used data from 9,295 adolescents aged 11 to 17 years from all nine regions in Austria. Of these adolescents, 7,948 pupils participated in the Austrian Health Behavior in School-Aged Children (HBSC) Study 2021/22 [22]. They visited five different Austrian school types covering junior high schools (*Mittelschule*), secondary schools (*Allgemeinbildende Höhere Schule*, *Berufsbildende Mittlere Schule*, *Berufsbildende Höhere Schule*) and polytechnical schools (*Polytechnische Schule*).

In addition, 1,345 apprentices participated in the Austrian Health Study on Apprentices 2021/22 [23]. In Austria, apprenticeship training includes simultaneous vocational training in companies and vocational schools and typically lasts three years. In this sample, 48.7% of the apprentices were in their first year of training, 40.8% in their second and 10.5% in their third.

Overall, the sample covers 9.2% 11-year-olds, 7.5% 12-year-olds, 12.0% 13-year-olds, 13.3% 14-year-olds, 22.3% 15-year-olds, 16.8% 16-year-olds and 19.0% 17-year-olds, and 21.2% had a migration background. The ratio of pupils and trainees (85.5% to 14.5%) in the sample resembles the ratio (84.8% to 15.2%) in the Austrian population. However, the age distribution deviated from the distribution of adolescents in the Austrian population (varying between 13.5% and 14.7% in the included age groups) [24].

### Design

The HBSC study is an international online survey conducted anonymously in 51 countries and regions every four years with a special focus on the health and health behavior of adolescents. Data were collected using an international standard questionnaire that participating adolescents filled out. The Austrian Health Study on Apprentices used an abbreviated HBSC study protocol but with additional items on work-related topics. Recruitment and data collection of both studies took place between October 11, 2021, and July 9, 2022. The study team invited all schools of the relevant school types to take part in the study via e-mail. Only classes of grades 5, 7, 9, and 11 were allowed to participate, with a maximum of two classes per school. In vocational schools, the participation of apprentices in their second year of training was facilitated.

### Measures

The main measures of this study were two questions concerning gender identity and sexual orientation. In previous iterations of the HBSC survey, gender identity was originally measured with a single-choice item presenting the statement “Your gender [Dein Geschlecht]:” and the answer options “male [männlich]” and “female [weiblich]”. This item was carefully expanded in the wave of 2021/2022 in Austria with a third answer option “other, and that is [anderes, und zwar]: ______”. The reasons for this format were (1) to gather information on other gender identities than male and female, (2) to allow descriptions to explore the diverse range of other gender identities, and (3) to stay as close to the previous format as possible. The chosen item, however, did not differentiate between cis- and transgender adolescents. Both cis- and transgender adolescents may find the answer options “male” or “female” suitable for themselves, because the German term “Geschlecht” is used for both, sex and gender.

Sexual orientation was measured for the first time in 2021/22 and used a single-choice item that was only presented to adolescents aged 14 to 17 years: “How do you describe your sexual orientation?”. The answer options included “heterosexual”, “bisexual”, “gay or lesbian”, “other, and that is: _____” and “I am not sure (yet)”. Again, the other option allowed for further descriptions to explore the diverse range of other sexual orientations.

### Data analysis

First, we calculated sample weights on the basis of age and sex to improve the generalizability of the results for Austrian adolescents [24]. We conducted all following analyses with and without sample weights and no meaningful differences occurred. Differences were only found in the second decimal place. To reduce unnecessary complexity, we omitted the sample weights, and the results can be interpreted as representative of the Austrian population of adolescents aged 11 to 17 years, nevertheless.

In the second step, two authors categorized the open answers given as specific gender identities or sexual orientations. If these answers did not represent gender identities or sexual orientations they were further categorized as mockery, inconclusive answers, or rejections (when the participants did not want to tell us this information).

For the first and second research questions, we conducted descriptive analyses showing the distributions of gender identities and sexual orientations separately for age groups and, exploratorily, pupils vs. apprentices. We also calculated distributions of sexual orientations within the different gender identities. Additionally, we conducted two Chi²-tests for gender identity and sexual orientation to investigate whether the distributions varied significantly by age or exploratory by group (pupils vs. apprentices). For these Chi²-tests we dichotomized gender identity in *other* versus *male/female* and sexual orientation in *bisexual/homosexual/other* versus *heterosexual*. Furthermore, we conducted one more Chi²-test to investigate whether the proportion of adolescents who responded to be not sure about their sexual orientation differed with respect to age. For this Chi²-test we dichotomized sexual orientation in being *not sure* vs. *heterosexual/bisexual/homosexual/other*. In the last step, we analyzed the distributions of sexual orientations within the different gender identities and conducted a Chi²-test to identify possible differences.

For the third research question, we categorized the mocking answers given on both items as topics following a qualitative content analysis [25]. We conducted descriptive analyses separately for gender identity and sexual orientation and provided examples for each topic.

## Results

### Categorization of open answers

Regarding gender identity, a total of 114 (1.2%) of the 9,295 adolescents used the “other” answer option and were allowed to further specify their gender identity. Of these adolescents, 38 (0.4%) used expressions that represented gender identities (e.g., nonbinary, agender, transgender, genderfluid) and 36 (0.4%) gave mocking answers. The remaining answers consisted of inconclusive or missing explanations, as well as not being sure about one’s own gender identity or rejections. Table 1 shows the distribution of the specifications of gender identity along with examples.

**Table 1:** Categories, distribution, and examples of the specifications on gender identity.

Category	*n*	%*	Examples
Agender	3	0.0	–
Transgender	5	0.1	–
Genderfluid	3	0.0	–
Nonbinary	27	0.3	–
Rejection	2	0.0	I will not tell you
Not sure (yet)	5	0.1	Still finding it out, having no clue yet, just do not know
Inconclusive	14	0.2	Diverse, human, first names
Missing	19	0.2	–
Mockery	36	0.4	Cat, cheese, alien, combat helicopter
Total	114	1.2	–

* Percent in relation of total sample (n = 9.295)

More adolescents, i.e., 187 (3.0%) of 6,127, chose the “other” answer option when asked about their sexual orientation. The descriptions of 109 adolescents (1.8%) represented various sexual orientations (e.g., pansexual, asexual, queer, demisexual, polysexual). Moreover, 46 (0.8%) mocking answers were given, and 32 (0.5%) answers were inconclusive or missing. Table 2 shows the distribution of the specifications of sexual orientation along with examples.

**Table 2:** Categories, distribution and examples of the specifications on sexual orientation.

Category	*n*	%*	Examples
Asexual	11	0.2	–
Pansexual	76	1.2	Pan, omnisexual
Demisexual	4	0.1	–
Queer	6	0.1	–
Polysexual	4	0.1	–
Other	8	0.1	No label, sapiosexual, abrosexual
Inconclusive	15	0.2	Humans, pride, gender, allrounder, first names
Missing	17	0.3	–
Mockery	46	0.7	Monkey, toast, cool, optimus prime
Total	187	3.0	–

* Percent in relation of total sample (n = 6.146)

### Distribution of gender identities

Most participating adolescents identified themselves as either male (44.1%) or female (54.7%). However, 0.8% had another gender identity with nonbinary being the most common choice. Only five adolescents (0.1%) were not sure about their gender identity and 0.4% gave mocking answers. Table 3 shows the distributions of gender identities for different age groups and pupils vs. apprentices. The Chi²-tests did not yield significant results, showing that gender identity did not vary with respect to age (*χ²*_(6)_ = 7.24, *p* =.150) or between pupils and apprentices (*χ²*_(1)_ = 0.04, *p* = .847).

**Table 3:** Distribution of gender identities in Austrian adolescents by age and group (pupils vs. apprentices).

	Male		Female		Other		Unsure		Mockery	
	*n*	%	*n*	%	*n*	%	*n*	%	*n*	%
11-year olds	418	49.1	430	50.5	1	0.1	0	0.0	2	0.2
12-year olds	329	47.0	366	52.3	4	0.6	0	0.0	1	0.1
13-year olds	523	47.0	575	51.7	10	0.9	0	0.0	5	0.4
14-year olds	531	43.1	683	55.4	11	0.9	3	0.2	4	0.3
15-year olds	882	42.6	1.158	55.9	18	0.9	0	0.0	13	0.6
16-year olds	681	43.6	865	55.4	11	0.7	0	0.0	4	0.3
17-year olds	735	41.6	1.005	56.9	18	1.0	2	0.1	7	0.4
Pupils	3.371	42.4	4.479	56.4	63	0.8	4	0.1	31	0.4
Apprentices	728	54.0	603	44.8	10	0.7	1	0.1	5	0.4
Total	4.099	44.1	5.082	54.7	73	0.8	5	0.1	36	0.4

### Distribution of sexual orientations

Around 4 out of 5 adolescents identified themselves as heterosexual (82.9%) and every tenth adolescent identified with another sexual orientation (10.5%), followed by 5.8% of adolescents who were not sure about their sexual orientation yet, and finally, 0.8%, who gave mocking answers. Within the other sexual orientations, bisexuality was the most common orientation (6.7%), followed by homosexuality (1.5%) and pansexuality (1.2%).

Table 4 shows the distribution of sexual orientations for different age groups and pupils vs. apprentices. The first two Chi²-tests did not yield significant results, showing that sexual orientation did not vary with respect to age (*χ²*_(3)_ = 1.68, *p* = .320) or between pupils and apprentices (*χ²*_(1)_ = 0.31, *p* = .580). However, a trend was identified with the third Chi²-test showing that younger adolescents were more often unsure about their sexual orientation (*χ²*_(3)_ = 5.73, *p* = .063) than older adolescents. In detail, from age 14 to 17, the proportion of adolescents who were unsure reduced from 6.7% to 5.1%.

**Table 4:** Distribution of sexual orientations in Austrian adolescents by age and group (pupils vs. apprentices).

	Hetero sexual		Bisexual		Homosexual		Other		Not sure		Mockery	
	*n*	%	*n*	%	*n*	%	*n*	%	*n*	%	*n*	%
14-year olds	828	83.2	68	6.8	9	0.9	16	1.6	67	6.7	7	0.7
15-year olds	1.612	81.8	123	6.2	39	2.0	52	2.6	128	6.5	16	0.8
16-year olds	1.251	83.7	94	6.3	22	1.5	41	2.7	78	5.2	8	0.5
17-year olds	1.389	83.3	124	7.4	23	1.4	32	1.9	85	5.1	15	0.9
Pupils	4.013	82.4	331	6.8	73	1.5	110	2.3	307	6.3	35	0.7
Apprentices	1.067	84.8	78	6.2	20	1.6	31	2.5	51	4.1	11	0.9
Total	5.080	82.9	409	6.7	93	1.5	141	2.3	358	5.8	46	0.8

**Fig 1:**
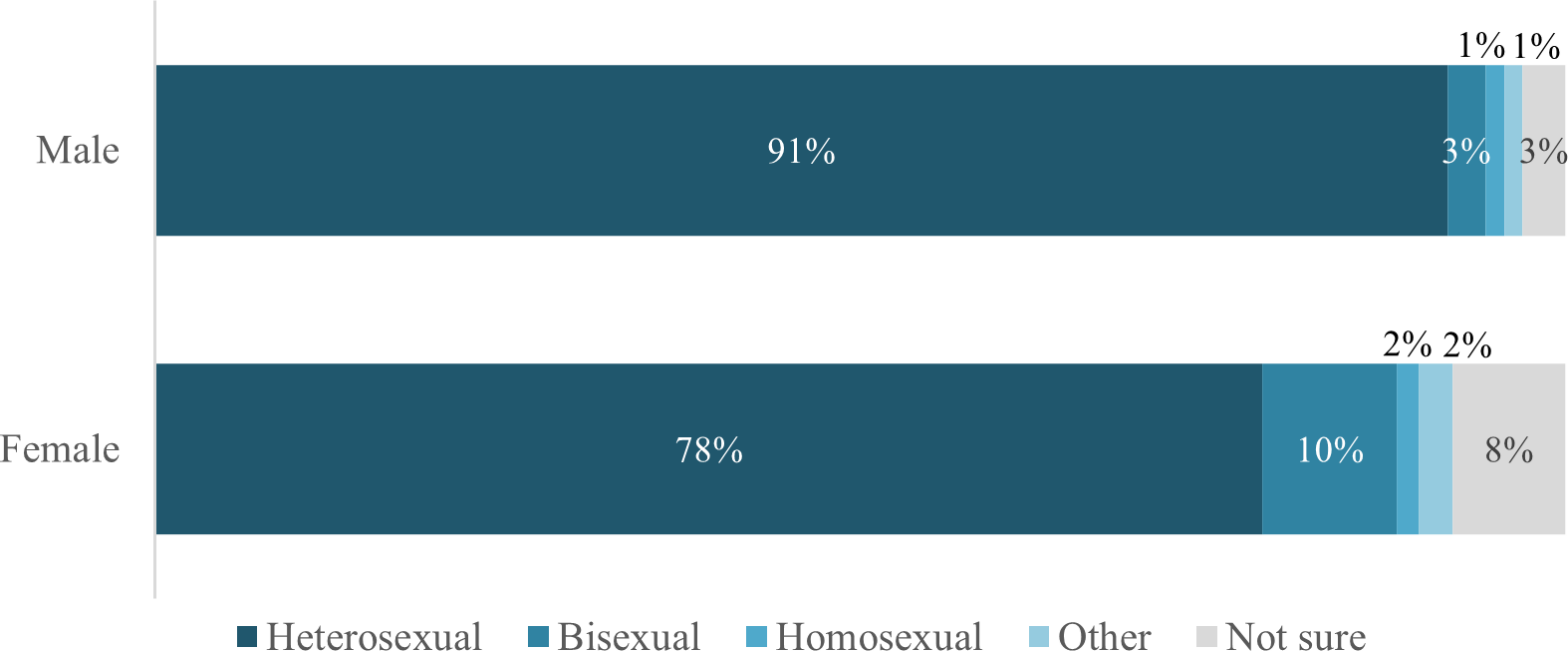
Distribution of sexual orientations in male and female Austrian adolescents (mocking answers excluded).

We originally planned to analyze the distribution of the different sexual orientations for all gender identities. However, the group size of adolescents with other gender identities was too small to yield trustworthy results, especially when split into subgroups. Therefore, we analyzed the distributions for male and female adolescents only (Fig 1). The corresponding Chi²-test was significant (*χ²*_(20)_ = 1069,12, *p* < .000), showing that the proportion of heterosexual adolescents was higher in male (90.7%) than female adolescents (78.3%) and all other sexual orientations were more common in females than males. That is, 10% of female and 3% of male adolescents identified as bisexual, 2% of females and 1% of males identified as homosexual as well as other orientations. Furthermore, the proportion of adolescents who were unsure about their sexual orientation was higher in females (8%) than in males (3%).

### Mocking answers

**Fig 2:**
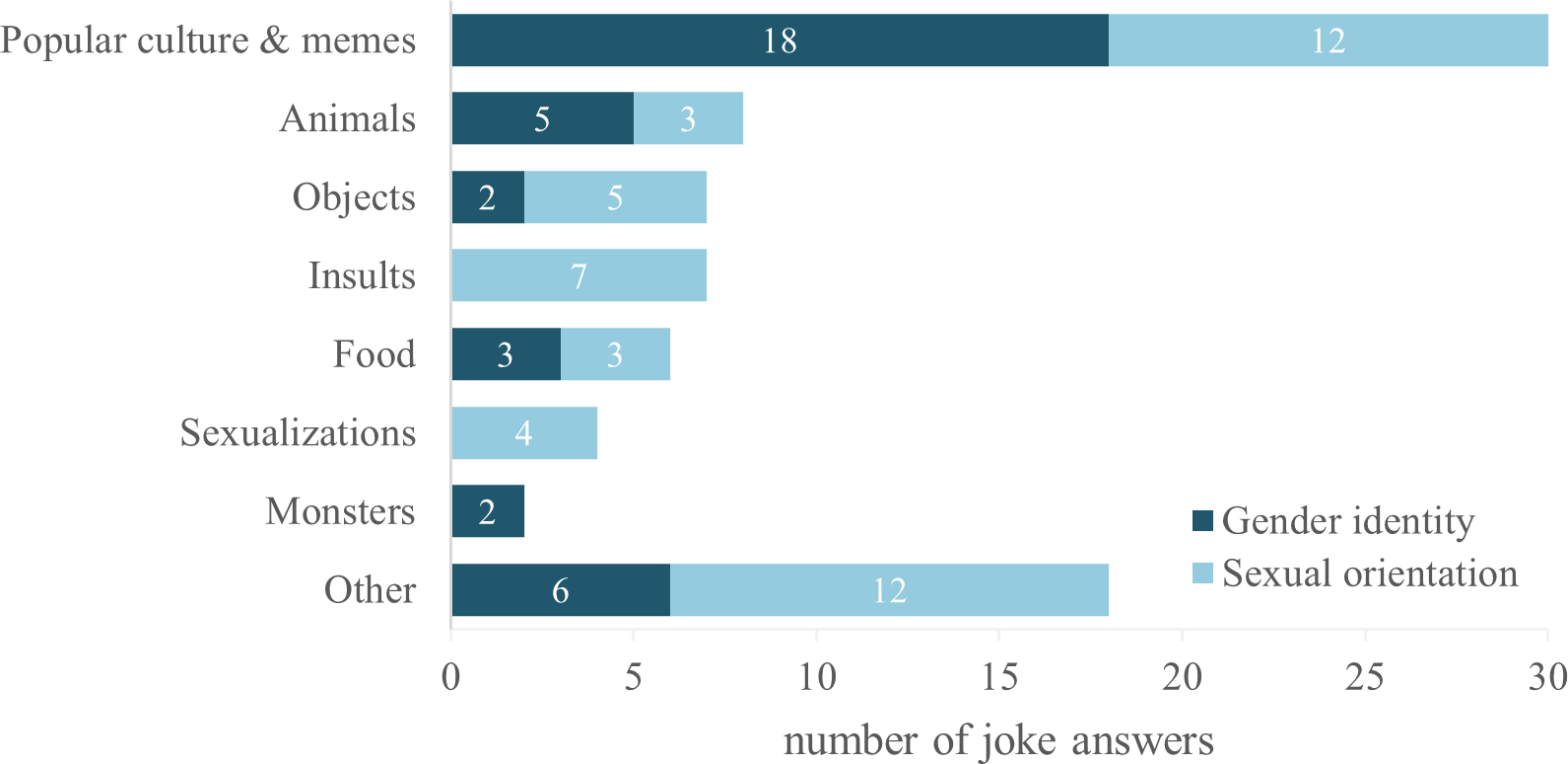
Categories of mocking answers by gender identity and sexual orientation.

In total, 82 mocking answers were given. After we performed three iterations of qualitative categorization, we identified seven topics and assigned 64 answers to them (Fig 2). Eighteen answers (e.g., cool, Pastafarian, Venezuela) could not be categorized in a meaningful way. Mocking answers were more often received for the question of sexual orientation than of gender identity. Most of these fell under the topic *Popular Culture & Memes* (e.g., BTS, transformer, Pokémon), and “combat helicopter” was the most common answer overall. The topics *Animals* (e.g., monkey), *Objects* (e.g., processor, panflute), *Insults* (e.g., your mother), and *Food* (e.g., donut, toastbread) were used almost equally. Interestingly, *Insults* and *Sexualizations* (e.g., flesh light) only occurred when asked about sexual orientation. The least common category was *Monsters* (e.g., alien, troll) and occurred exclusively when asked about gender identity.

## Discussion

This study is among the first European studies that gathered data on country-wide proportions of LGBTQ youth. The results show that at least 0.8% of Austrian adolescents aged 11 to 17 years identified with a gender other than cisgender. Nonbinary was the most common gender identity within that group. Additional, self-identified labels included agender, genderfluid, and transgender. Due to the lack of distinct classifications of transgender youth in our surveys, we expect the actual proportion of non-cisgender adolescents to be higher in Austria. In other European countries, the upper bounds of the estimates hit 1.6% to 4.6% [8–10,13] and Austria should not differ severely.

Further, 10.5% of Austrian adolescents identified with a sexual orientation other than heterosexual, with bisexual being the most common, followed by homosexual. The specifications of other sexual orientations included pansexual, asexual, queer, polysexual, demisexual, sapiosexual, and abrosexual. In the large-scale survey [13] in 27 countries the average estimate was nine percent in total, and eleven percent in Hungary and Germany. The German survey on sexual orientation in youth [12] showed that six to seven percent of 14- to 17-year-olds identified as homo- or bisexual.

From a developmental psychological perspective, our data shows that adolescents who are unsure about their gender identity are rare and homogeneous in our age group. This is in line with findings in Medico et al. [14] and Scandurra et al. [15] showing that the onset of puberty marks a common time point to address and/or intensify the exploration of feelings of gender dissonance. Moreover, as being unsure was very seldom in our sample, this time point may be suitable for other gender than transgender identities (e.g., genderfluid, nonbinary) as well. Regarding sexual orientation, the proportion of adolescents being unsure was higher, underlining that the development of one’s sexual orientation takes longer for some adolescents [17]. However, the proportion decreased between 14- and 17-year-olds.

We further analyzed whether sexual orientations vary between girls and boys and found that heterosexuality is more common in Austrian boys than girls. Girls were more often unsure about their sexual orientation or bisexual, compared to boys. This is in line with the study on developmental milestones of sexual orientation, showing that, on average, cisgender girls need two years longer than cisgender boys to Self-Identify [18]. The gender difference in bisexuality is also found in other studies on young people, [e.g., 12,13]. One explanation for this difference is that Western societies might be more tolerant towards bisexuality in cisgender women than in cisgender men [26].

Exploratory analysis revealed that there were no differences in gender identity and sexual orientation between pupils and apprentices, underlining that they are independent of educational background [see also 13]. This fact indicates independence regarding sociodemographic background, as adolescents from lower-income families are more often found to take up apprenticeships than those from higher-income families [27].

Mocking answers were infrequent and, as expected, most of them represented ideas and concepts from popular culture. The most used mockery was “combat helicopter”, a meme that stems from a text written by a gamer of a multiplayer war game in 2014. As soon as somebody posted a comment about gender identity in the game’s chat, he responded with a text where he identified as a combat helicopter, mocking any gender identification other than cisgender [28]. Surprisingly, this meme persists even ten years later on the internet and the minds of today’s adolescents, underlining the role of past and present popular culture in the development of attitudes in youth.

Another phenomenon we found is that some mocking answers resemble valid labels for LGBTQ people. For example, we found that “TRANSformer” and “PANflute” were used. This shows that adolescents may associate certain labels with figures and objects that sound similar or share certain letters. Additionally, mockeries from the Monster topic were only found regarding gender identity. From this, it seems that certain adolescents do not have enough information on gender identities other than cisgender or tend to mystify (e.g., an alien with unknown, superhuman powers) the fact that people may identify with another gender than they were assigned at birth. Insults, on the other hand, were only found in answers on sexual orientation. This may be explained by many swear words and phrases in Austria that still make use of labels of other sexual orientations than heterosexual, reproducing attitudes that see them as bad and inferior.

Methodologically, the data loss due to mocking and inconclusive answers was around half a percent when focusing on the item on gender identity. Since gender identity is a variable that is often used as a grouping variable, we need to weigh the costs and uses for including more gender identity options than male and female. We argue that the loss is negligible as the sample sizes of the male and female subgroups still exceed the requirements for descriptive and inferential statistics. Further, adolescents with other gender identities than cisgender are found to be at higher risk for mental health problems as well as socio-emotional stress, [e.g., 21,29–31], and corresponding research is still needed.

However, the interpretation of the study results necessitates to consider some limitations. In our surveys, the items on gender identity and sexual orientation do not follow the current recommendation of state-of-the-art measurements [8,9,32] of these constructs. This led to issues with the gender identity item, as it did not allow clear classifications of transgender youth and will need a rework in further survey waves. The HBSC study team is already working on a better solution in this regard. Additionally, the text fields to specify “other”-options on both items were presented simultaneously and therefore may have provoked mocking answers in some adolescents. We advise, if technically possible, to split the text fields from the other parts of the item and to present it only if the “other”-option was chosen and on the next page of the survey.

Further limitations concern the small group sizes of gender identities other than cisgender, which did not allow quantitative analyses to be conducted separately for these subgroups. However, these group sizes are common in other countries and adulthood as well, [e.g., 8,13]. Additionally, the sample was not fully representative of Austria’s youth, and we calculated sample weights to correct for that. As we conducted the analyses with and without sample weights and found no meaningful differences in the results, we omitted the sample weights to reduce unnecessary complexity. Lastly, as gender identity and sexual orientation are still an ongoing development process during the period of puberty and young adulthood [33,34], and self-labelling may also shift while transitioning in transgender youth [35], the interpretation of our results should be taken with caution. These proportions are a snapshot, and some of the participating adolescents may change their labels (several times) in the next years.

First and foremost, this study shows that gathering data on LGBTQ youth is, from a developmental perspective, age-appropriate for 11- to 17-year-olds. In Austria, more than 10% of current youth are LGBTQ. An overwhelmingly large part (over 99%) of the participants took the questions on gender identity and sexual orientation seriously. Therefore, more surveys in various countries and regions, [e.g., 36] should consider including comprehensive items on gender identity and sexual orientation. This gives LGBTQ youth the opportunity to report on their current situation and needs, providing useful data for policymakers to raise awareness as well as planning and monitoring corresponding projects and initiatives.
